# Post-embolization Syndrome Following Yttrium-90 Radiation Segmentectomy

**DOI:** 10.7759/cureus.35947

**Published:** 2023-03-09

**Authors:** Naisarg B Vanani, Abhishek Janardan, Nisar Asmi, Pinky Jha

**Affiliations:** 1 Medicine, Medical College of Wisconsin, Milwaukee, USA; 2 Internal Medicine, Medical College of Wisconsin, Milwaukee, USA

**Keywords:** hepatocellular carcinoma, radiation oncology complication, tare complication, interventional radiology, 90-y radiation segmentectomy, hepatocellular carcinoma (hcc), transarterial radioembolization, post-embolization syndrome

## Abstract

Post-embolization syndrome (PES) is a complication that commonly occurs after treatment with transarterial embolization for hepatocellular carcinoma (HCC). Patients with PES often present with clinical symptoms such as fever, nausea, abdominal pain, and elevated liver enzymes typically 24-72 hours after the procedure. While cases of PES have been documented in patients treated with transarterial chemoembolization, here, we present an unusual case of delayed onset PES in a 70-year-old male with HCC following treatment with a form of transarterial radioembolization.

## Introduction

Hepatocellular carcinoma (HCC) is considered the leading cause of mortality in patients with liver cirrhosis, with trends in the US showing consistently increasing incidence and mortality, with a projected 33.9% increase in mortality projected by 2040 [1]. Treatments for HCC are often guided by the Barcelona Clinic Liver Cancer (BCLC) staging, which indicates transarterial chemoembolization (TACE) or transarterial radioembolization (TARE) as a treatment option, especially for BCLC class B patients [2,3].

Post-embolization syndrome (PES) is a complication that has been reported in patients after treatment with transarterial embolization procedures for HCC, typically presenting with symptoms such as fever, nausea, abdominal pain, and fatigue within 72 hours post-procedure. Compared to PES following TACE, there is a relative paucity of PES and lower instances of hospitalization following TARE [4]. PES following TACE has been attributed to inflammatory pathophysiology due to tumor necrosis and ischemia to healthy liver parenchyma, resulting in PES in 60-80% of cases [5,6]. For this reason, patients are admitted for observation post-TACE procedure to manage any PES symptoms that arise. While symptoms of PES are generally self-limited, prophylactic N-acetylcysteine and dexamethasone-lipiodol treatments have been demonstrated to reduce PES after TACE procedures [7,8].

In the case of TARE procedures, radioembolization does not result in immediate tissue ischemia like TACE but can cause gradual inflammation due to the effects of radioactivity in the parenchyma surrounding the therapeutic target, offering a possible explanation of the rarity and relative mildness of PES following TARE [9]. Due to the variable presentation of PES following TARE procedures and lack of post-procedural inpatient monitoring or prophylactic treatments, it is important to be aware of the possibility of later onset with a broader range of symptom severity in post-TARE PES, which could necessitate inpatient symptomatic management. Here, we present a case of a 70-year-old man with a history of HCC and underlying cirrhosis with portal hypertension who presented with an unusual, late-onset PES eight days post-yttrium-90 (90Y) radiation segmentectomy, representing the first case report to present PES following 90Y radiation segmentectomy.

## Case presentation

**Figure 1:**
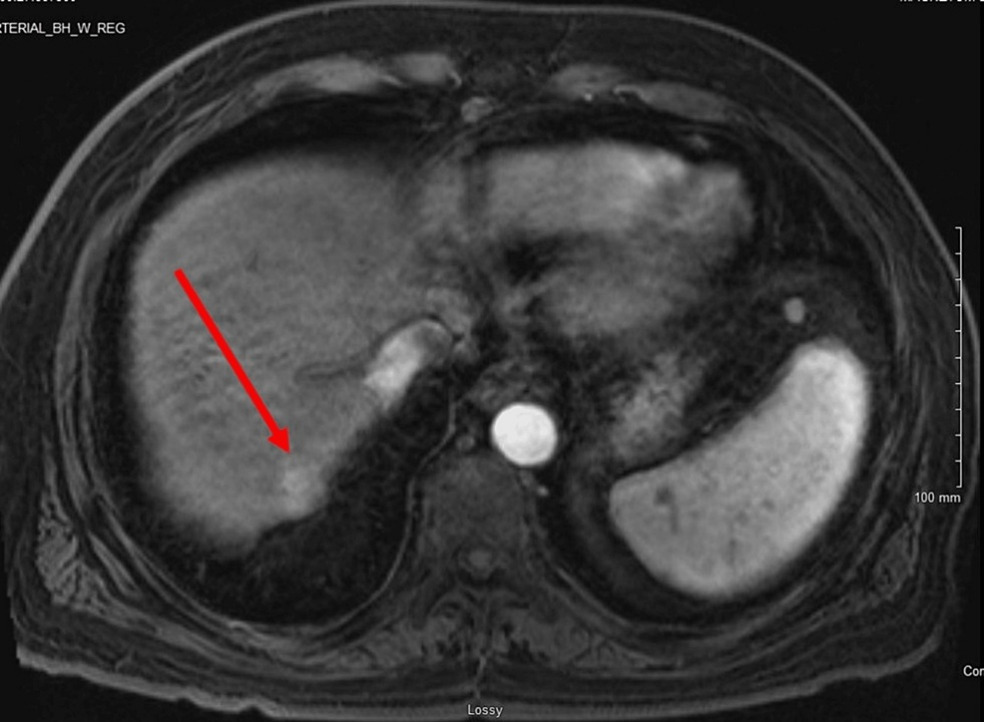
MRI of segment VII lesion MRI of the segment VII hepatocellular carcinoma lesion observed (arrow) with arterial phase enhancement.

A 70-year-old Hispanic male with a history of HCC, non-alcoholic steatohepatitis (NASH) cirrhosis, portal hypertension, esophageal varices, diabetes mellitus, and hypertension presented to the emergency department (ED) with complaints of a high fever of 102°F (38.9°C) and nausea for two days. The patient had undergone a TARE of a segment VII HCC lesion using 90Y radiation segmentectomy eight days prior to arrival. The latest magnetic resonance imaging (MRI) prior to this TARE procedure had shown a 1.9 cm lesion in segment VII, which prompted this use of 90Y radiation segmentectomy (Figure 1).

On arrival at the emergency department, the patient’s vitals indicated a blood pressure of 183/80 mmHg, a pulse of 75 beats/minute, a body temperature of 100.5°F (38.1°C), a respiratory rate of 18 respirations/minute, and oxygen saturation of 98%. Labs indicated thrombocytopenia with a nadir of 94*10^3/uL, mildly elevated lactate dehydrogenase (LDH) of 257 U/L, and were negative for leukocytosis. The patient's elevated liver enzymes with alkaline phosphatase (ALP) of 174 U/L, alanine transaminase (ALT) of 102 U/L, and aspartate transaminase (AST) of 72 U/L, compared to previous values (ALP = 126 U/L, ALT = 75 U/L, and AST = 65U/L) prompted imaging studies of the liver and gallbladder. Additionally, given the patient’s active cancer, recent interventional radiology procedure, and a two-day history of high fevers and presenting vital signs, there was initial concern for bacteremia. Due to this initial concern for infectious processes and fevers of unknown origin, the patient was started on cefepime and metronidazole pending further lab and imaging studies.

**Figure 2:**
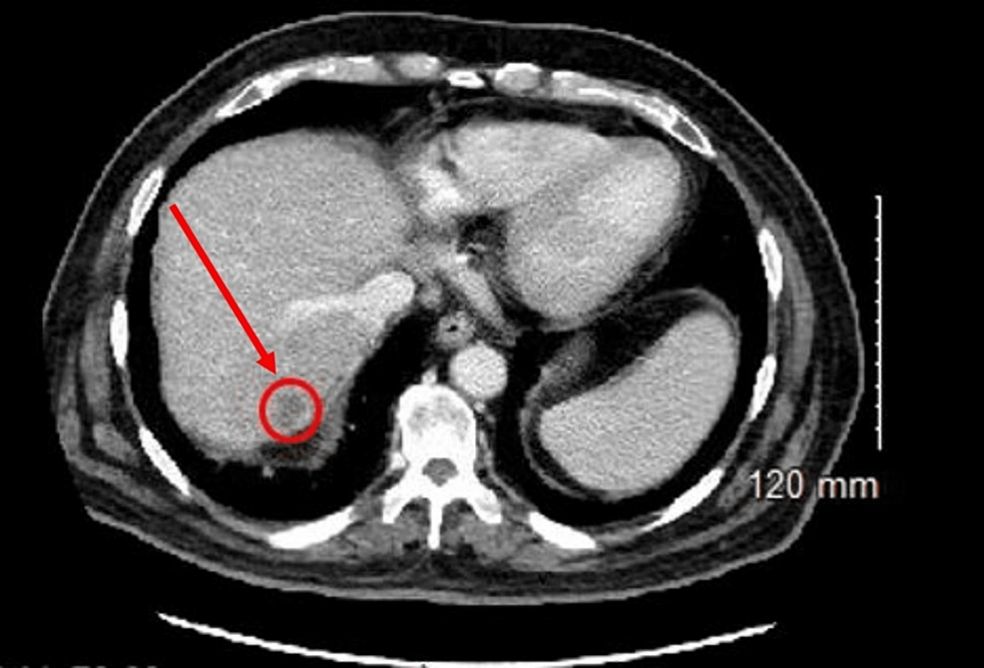
Axial CT of the abdomen and pelvis Axial CT of the abdomen and pelvis visualizes the hypodense lesion previously treated by 90Y segmentectomy (encircled in red) in hepatic segment VII with some peripheral enhancement.

**Figure 3:**
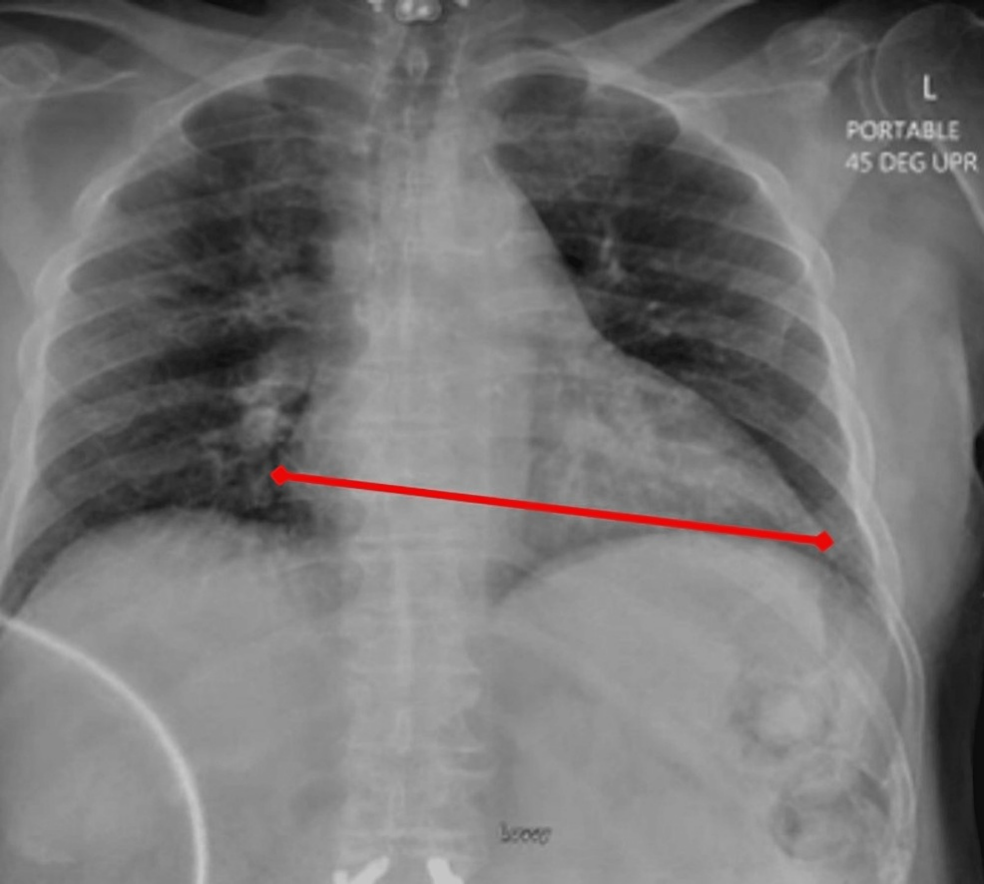
Chest radiograph (anteroposterior view) - hospital day two Cardiomegaly (red line) and diffuse interstitial coarsening are visualized on the chest radiograph.

Computed tomography (CT) imaging indicated cholelithiasis and the presence of subtle pericholecystic fat stranding, with no significant gallbladder lumen distention. Additionally, the CT scan showed no signs of active infection of the liver or gallbladder, and the presence of a hypodense lesion in segment VII compatible with the recently treated HCC was visualized (Figure 2). This was followed up by an upper quadrant ultrasound (US) to further evaluate for cholecystitis, which resulted as negative with the absence of any wall thickening, pericholecystic fluid, hyperemia, and significant luminal distention. Additionally, US findings were consistent with the CT scan for cholelithiasis but were negative for the sonographic Murphy sign, ruling out acute cholecystitis. The chest radiograph (CXR) confirmed that there were no findings of pulmonary infection, consolidation, effusion, or pneumothorax. While cardiomegaly and diffuse interstitial coarsening were visualized on CXR, this was similar to imaging six months prior (Figure 3).

The patient’s temperature remained elevated during their hospitalization, reaching a maximum of 103°F (39.4°C) on hospital day two before subsiding on hospital day three. Additionally, the blood cultures resulted as negative with no growth of infectious species throughout the course of hospitalization. Since this hospitalization and symptoms follow a recent 90Y radiation segmentectomy procedure, a diagnosis of fevers and elevated liver enzymes secondary to PES was made. The patient was treated with acetaminophen as needed for PES symptoms and was discharged home on hospital day four in stable condition.

## Discussion

This is a case of a 70-year-old male with a history of HCC and NASH cirrhosis presenting with high fevers, nausea, and elevated liver enzymes one week following a 90Y radiation segmentectomy of a segment VII hepatic lesion. Infectious etiology was ruled out during the hospitalization course, which demonstrates the possibility of this unusual and delayed onset presentation of PES in patients treated with TARE using the radiation segmentectomy approach.

PES has been a well-documented phenomenon experienced by patients specifically after TACE treatment typically presenting with symptoms such as abdominal pain, nausea, fever, and elevated liver enzymes 24-72 hours post-procedure. As 60-80% of patients treated with TACE experience some form of PES, a brief period of hospitalization is typically indicated for patients who have undergone TACE for monitoring [5]. However, TARE with 90Y presents itself as another option for the treatment of HCC, which was used in our case. As patients are not held for observation post-TARE, post-radioembolization syndrome has not been as uniformly documented as the PES seen following TACE [4,5]. The patient, in our case, had undergone a 90Y TARE procedure and experienced the first signs of PES six days (~144 hours) post-procedure, which is markedly different from the typical 24-72 hours timeframe of presentation post-operation. This delayed onset presentation in our case could be attributed to the microembolic mechanism of 90Y segmentectomy using glass microspheres, which does not involve immediate tissue ischemia, which is seen in the macroembolic mechanism of TACE [4,6,9]. For these reasons, this case represents an unusual presentation of PES and is the first case report to describe PES following a 90Y-radiation segmentectomy.

It is important to discuss the various forms of intra-arterial therapies used for the treatment of HCC. At a general level, intra-arterial therapies such as TACE and TARE have two major aims: firstly, the delivery of a therapeutic agent or radiation, and secondly, to cause ischemia to the targeted tumor via vessel occlusion [10]. Specifically, TARE procedures involve a radioembolization approach where radioactive microspheres are injected in an intra-arterial fashion to confer a targeted treatment of the isolated tumor through the use of 90Y, which acts as a β-emitter [11,12]. Unlike TACE, which is often complicated with PES and requires inpatient hospitalization, TARE has been associated with a better quality of life and patient experience, as it can be performed in an outpatient setting [13]. More specifically, radiation segmentectomy using 90Y is indicated for the TARE treatment of small hepatic tumors less than 3 cm in diameter in a segmental manner [4,14]. While this can be achieved via the use of either glass microspheres or resin microspheres, a retrospective study of 90 patients indicated bilirubin and AST toxicities occurred at rates 2.8-fold and 2.6-fold higher in 90Y-resin microspheres compared to glass microspheres [14,15]. In our case, 90Y-glass microspheres were used to treat the patient's HCC tumor as a previous MRI had indicated a subcapsular 1.9 cm tumor in segment VII, which met the criteria for this form of TARE radiation segmentectomy.

Previous literature has attributed the lower hospitalization rate and PES associated with TARE procedures to the microembolic nature of 90Y treatment versus the macroembolic occlusion seen with TACE, predisposing TACE therapy to greater PES effects [13,16]. Typically pre-procedural angiography is conducted to identify and embolize any smaller vascular collaterals and connections to other extrahepatic tissues and organs, as was conducted with our patient. This serves to prevent some severe complications that have been shown to occur due to irradiation of extrahepatic tissues, which has resulted in complications such as gastrointestinal ulcerations, radiation pneumonitis, and cholecystitis [17,18]. In a study of 515 patients treated with a total of 680 TARE treatments using 90Y-resin microspheres, it was found that 4% of these treatments resulted in radioembolization-induced liver disease (REILD). Furthermore, 75% of these cases were from a single center, suggesting an operator-dependent effect [19]. It is also important to note that due to their partial embolic effects, 90Y-resin microspheres have been shown to have higher rates of post-radioembolization syndrome with regard to both REILD and extrahepatic complications when compared to 90Y-glass microspheres [4,20]. An important measure used to quantify laboratory hepatic toxicity is AST toxicity, ranging from grade 0-4/4, with a grade 3/4 constituting >5.0-10x the upper limit of normal AST value [21]. Specifically, in a retrospective review of 90 patients treated with 90Y-microspheres (21 resin, 69 glass), a grade 3/4 AST toxicity was observed in 39% of patients treated with 90Y-resin microspheres, compared to 9% in those treated with 90Y-glass microspheres [15]. This further highlights the rarity of our case of PES with a deranged liver profile after treatment with 90Y-glass microspheres and emphasizes the importance of awareness of this rare but serious complication profile of TARE.

## Conclusions

This case report highlights the possibility of an unusual and variable profile of PES following TARE procedures compared to the classic presentation following TACE. With TARE becoming a more popular option for the treatment of HCC, and increasingly offered as an outpatient treatment without post-procedure observation, similar presentations to our case may occur and go unchecked without sufficient patient follow-up. Thus, it is essential for physicians to be wary of delayed-onset PES and educate patients on the possibility of a late-onset PES that could necessitate further evaluation and hospitalization for symptomatic management and exclusion of infectious etiology in immunocompromised patients.
